# Identification of Risk Factors Affecting Impaired Fasting Glucose and Diabetes in Adult Patients from Northeast China

**DOI:** 10.3390/ijerph121012662

**Published:** 2015-10-12

**Authors:** Yutian Yin, Weiqing Han, Yuhan Wang, Yue Zhang, Shili Wu, Huiping Zhang, Lingling Jiang, Rui Wang, Peng Zhang, Yaqin Yu, Bo Li

**Affiliations:** 1 Department of Epidemiology and Biostatistics, School of Public Health, Jilin University, 1163 Xinmin Street, Changchun, Jilin 130021, China; E-Mails: yinyutian1123@126.com (Y.Y.); hanwq1987@foxmail.com (W.H.); 626699shiwo@163.com (Y.W.); ruiwen9090@126.com (Y.Z.); jianglingling.2008@163.com (L.J.); yj2002___yj@163.com (R.W.); w1099358429@sohu.com (P.Z.); yuyaqin5540@163.com (Y.Y.); 2 Administration Bureau of Changbai Mountain Natural Mineral Water Source Protection Areas, Jilin 130021, China; E-Mail: 5813091@163.com; 3 Department of Psychiatry, Yale University School of Medicine, New Haven, CT 06511, USA; E-Mail: huiping.zhang@yale.edu

**Keywords:** impaired fasting glucose (IFG), type 2 diabetes mellitus (DM), blood glucose, and hair metallic elements, polytomous logistic regression

## Abstract

*Background*: Besides genetic factors, the occurrence of diabetes is influenced by lifestyles and environmental factors as well as trace elements in diet materials. Subjects with impaired fasting glucose (IFG) have an increased risk of developing diabetes mellitus (DM). This study aimed to explore risk factors affecting IFG and diabetes in patients from Northeast China. *Methods*: A population-based, cross-sectional survey of chronic diseases and related risk factors was conducted in Jilin Province of Northeast China. All adult residents, aged 18–79, were invited to participate in this survey using the method of multistage stratified random cluster sampling. One hundred thirty-four patients with IFG or DM and 391 healthy control subjects were recruited. We compared demographic factors, body size measurements, healthy-related behaviors, and hair metallic element contents between IFG/diabetes patients and healthy individuals. *Results*: IFG/diabetes patients had a greater weight, waist, hip, and body mass index (BMI) than control subjects. Significant differences in the content of zinc (Zn), potassium (K), copper (Ca), and sodium (Na) as well as Cu/Zn ratios between IFG or DM patients and control subjects (*p* < 0.05) were also observed. Hair Cu, selenium (Se), and Na contents were positively correlated with blood glucose levels (Cu: *r*_s_ = 0.135, *p* = 0.002; Se: *r*_s_ = 0.110, *p* = 0.012; Na: *r*_s_ = 0.091, *p* = 0.038). Polytomous logistic regression adjusting for age, sex, family history of diabetes and BMI, showed that subjects with high BMI were more likely to develop IFG and DM (IFG: OR = 1.15, OR 95% CI = 1.02–1.29; DM: OR = 1.15, OR 95% CI = 1.01–1.33). Moreover, rarely or never eating fruits was a risk factor for DM (OR = 5.46, OR 95% CI = 1.87–15.98) but not for IFG (OR = 1.70, OR 95% CI = 0.72–4.02). Subjects with abdominal obesity or DM history were more susceptible to DM (abdominal obesity: OR = 2.99, OR 95% CI = 1.07–8.37; DM history: OR = 2.69, OR 95% CI = 1.01–7.20). We found that subjects living in Changling County had a significantly lower chance to suffer from IFG (OR and 95% CI for OR: 0.25, 0.08–0.74). Men and 60–69 years old subjects were at increased risk for IFG (male: OR = 3.51, OR 95% CI = 1.34–9.18; age 60–69: OR = 6.64, OR 95% CI = 1.36–32.47). We did not find significant associations of IFG or DM with certain lifestyles (such as eating more meat, exercise or physical activity, smoking, or alcohol drinking) or the content of some metallic elements (such as iron (Fe), Zn , K, calcium (Ca), Na, or magnesium (Mg)). *Conclusions*: This study demonstrated that less or no fruit eating, DM family history, abdominal obesity conferred vulnerability to DM. Living in Changling County, men and 60–69 years old subjects were found to be risk factors for IFG. Subjects with high BMI were more likely to develop IFG and DM.

## 1. Introduction

As one of the most serious chronic diseases, diabetes mellitus (DM) affects about 366 million people globally (8.3%), projection studies based on sample weighting indicate that by 2030, the number of DM patients may increase to 552 million (9.9%) [1]. In China, the estimated prevalence of diabetes and hyperglycemia was 11.6% and 50.1%, respectively, according to a cross-sectional survey among a nationally representative sample of 98,658 Chinese adults in 2010 [2]. In other words, there were 113.9 million Chinese adults suffering from diabetes and 493.4 million Chinese adults with hyperglycemia, and some of them may develop type 2 diabetes mellitus (T2DM) [1]. Similarly, there was a high prevalence in America, where diabetes was the seventh leading cause of death according to the data compiled from the US death certificates in 2007 [3]. Studies have speculated that the number of diabetes patients will increase to 29 million by 2050 in the US [4]. In Sweden, the number of patients with diabetes in 2012 was 390,000 [5]. A high prevalence of DM was also found in Jamaica (7.9%) [6]. In Portugal, the prevalence of DM in middle-aged and senior adults has increased 2–3 fold in the last two decades. In the same period of time, the mean fasting glucose level was increased 8 mg/dL and 7 mg/dL, respectively, in men and women [7]. These epidemiological studies indicate a growing medical burden of diabetes, particularly in developing countries [8].

In addition to health damages caused by diabetes itself, serious complications, such as heart diseases and stroke, nervous system disorders, diabetic nephropathy, and periodontal diseases [3], also appear in diabetes patients. The economic burden of diabetes to society is enormous. Disability-adjusted life years (DALY) for diabetes in China were 19.12 DALYs per 1000 subjects [9]. A recent study showed that in rural areas of Southwest China, the cost related to diabetes was up to $46.8 million and continues to increase due to the increasing prevalence of diabetes and the chronic nature of it [10,11,12,13]. As time continues to pass, diabetes will certainly become one of the most costly chronic diseases, and the burden of healthcare expenditures will continue to increase in China [14]. 

The fasting blood glucose level in impaired fasting glucose (IFG) individuals is consistently elevated above normal levels, but it is not high enough for DM diagnosis according to the Dorland’s Medical Dictionary. Subjects with IFG have an increased risk of developing diabetes, although many IFG individuals do not progress to diabetes. Lifestyle interventions (increasing physical exercise, lowering body mass and improving eating habits, *etc*.) are effective in delaying or even preventing the onset of diabetes [15].

In early 1990s, the American Diabetes Association (ADA) and the World Health Organization (WHO) defined IFG by fasting blood glucose levels (ADA: 5.6–6.9 mmol /L; WHO: 6.1–6.9 mmol /L) [16,17,18,19]. Although the standards for IFG from ADA and WHO are not consistent, IFG is commonly accepted as a predictor of T2DM [20,21,22]. If the criteria from ADA are used, the calculated prevalence of IFG would increase and may cause a series of question of overdiagnosis [16]. In this study, we used WHO’s diagnostic criteria to define IFG and diabetes. 

We recruited subjects aged 18 to 79 years old in representative regions (low prevalence of diabetes and/or considering the sampling results) of Jilin Province of Northeast China. Our study focused on subjects with IFG or diabetes, and the variables we studied included not only health factors, but also the concentrations of various metallic elements in hair. It could be a warning of disturbed glucose metabolism and insulin homeostasis if a person has IFG. At this stage, if risk factors for diabetes are controlled with lifestyle changes, blood sugar levels may gradually become normal or the onset of diabetes will be delayed. Existing evidence showed the importance of lifestyle interventions on diabetes prevention. There were three major trials performed in Finland (n = 522) [17], China (n = 577) [18], and the US (the Diabetes Prevention Program, n = 3234) [19]. Findings from these studies support that lifestyle interventions could reduce the incidence of diabetes over a several-year period. Although these interventions may not prevent the onset of diabetes, it is also very effective to delay the occurrence of complications [5]. Thus, studies of risk factors affecting IFG and diabetes as well as the intervention are important.

Besides genetic factors, the occurrence of diabetes is influenced by lifestyles and environmental factors as well as trace elements in diet materials. The homeostasis of trace elements in our body can be disrupted by IFG or diabetes. Disturbances of trace element uptake and metabolism may lead to insulin resistance and the development of diabetic complications. In addition, studies have shown that the content of elements in hair was associated with IFG and diabetes [20,21,22,23]. The aim of this study was to investigate whether geographical patterns, demographic factors, body sizes, health-related behaviors, and hair metallic element contents could affect IFG and diabetes in patients from Northeast China. Put forward some reasonable way of intervention according to the results of this study.

## 2. Experimental Section

### 2.1. Subjects

Demographic and clinical data were collected *via* a population-based, cross-sectional survey of chronic diseases and related risk factors in a population from Jilin Province (22,855,797 subjects in total, including 12,355,852 and 10,499,945 subjects in urban and rural areas, respectively [24]) of Northeast China. The method of multistage stratified random cluster sampling was used. At the first stage, the province was stratified into nine regions as per the existing administrative division (Changchun, Jilin, Siping, Liaoyuan, Tonghua, Baishan, Songyuan, Baicheng, and Yanbian). Considering the geographical locations and environmental and population differences, we chose three administrative regions (Liaoyuan, Baishan and Songyuan). At the second stage, a cluster of one county or district was randomly selected from each of the three regions using probability proportional to size (PPS) sampling. At the third stage, each selected county or district was divided into urban and rural areas as defined by the National Bureau of Statistics of China [24]. Subsequently, three or four communities were sampled from both urban and rural strata using PPS. Finally, one adult subject (aged 18–79) was randomly selected from each household of the selected communities. Participants were recruited through invitation or home visit. They were from DongLiao County of LiaoYuan, JingYu County of BaiShan, and ChangLing County of SongYuan in Jilin Province.

The inclusion criteria for the current study were: (1) adult (18 to 79 years old) residents from selected communities; and (2) voluntary participants in the survey. Subjects who were judged to be frail or ill were excluded from completing the survey.

**Figure 1:**
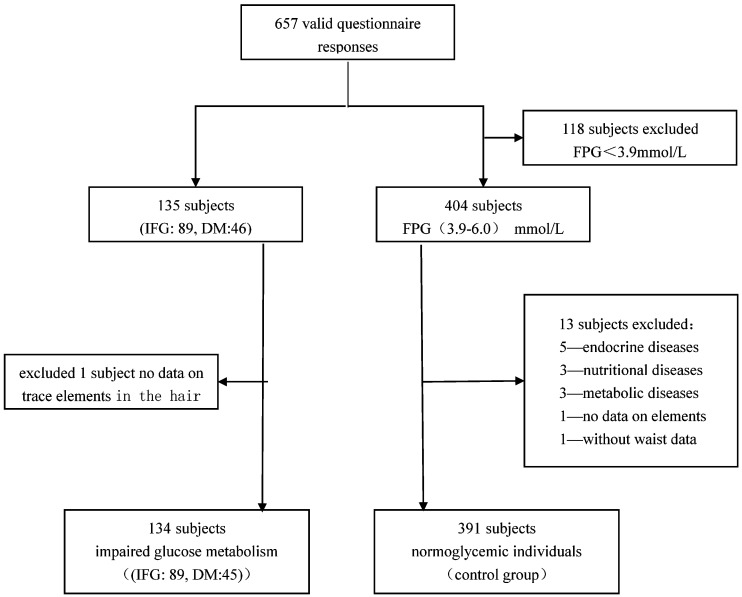
Flow diagram of patient recruitment.

A total of 657 valid questionnaire responses were received in this study. One hundred thirty-five subjects (89 with IFG, and 46 with DM) had fasting serum glucose levels more than 6.1 mmol/L, namely IFG/DM. Those subjects who self-reported having DM or being under treatment with either insulin or oral anti-diabetic agents were also included as diabetic patients. We excluded one subject with no data on trace elements in hair, geting 134 subjects with impaired glucose metabolism (IFG:89, DM:45). Some subjects with fasting serum glucose levels less than 3.9 mmol/L were also excluded (118). We excluded individuals affected with endocrine (5), nutritional (3), and metabolic diseases (3). One subject with no data on the content of metallic elements in hair and one subject without waist measurements were excluded. Fasting serum glucose levels in 391 control subjects ranged from 3.9 mmol/L to 6.1 mmol/L, without the factors that will exclude them from the analysis. Finally, 525 subjects (including 391 control and 134 impaired glucose metabolism subjects) were included in the data analysis. A flow diagram of patient recruitment was given in Figure 1.

### 2.2. Ethical Standards

The Ethics Committee of Jilin University School of Public Health approved this study (Reference Number: 2012-R-011). Written informed consent was obtained from each participant.

### 2.3. Data Collection

This survey consisted of three parts: questionnaire investigation (the demographic characteristics and health related behaviors, such as sex, age, and areas), body measurements (such as blood pressure, height, weight, and waist and hip circumferences), and laboratory measurements (blood glucose levels and the content of various metallic elements in hair). All investigators participating in this study had received uniform training, and they followed the same instruction of the questionnaire. The measurement instruments used were calibrated according to the same standard.

### 2.4. Measurements

The height and weight of subjects were measured without shoes, and body mass index (BMI) was calculated as the weight (kg) divided by the squared height (m^2^). The calibrated mercury sphygmomanometer was used to determine blood pressure of subjects on the right arm, after at least 5 min of seated rest. Blood pressure, including systolic and diastolic, was measured three times with intervals of at least one minute, and the average value of three readings was used for data analysis [25].

Waist circumferences were measured at the midpoint of the horizontal connections between the anterior superior iliac spine and the edge of 12 ribs on each side, *i.e.*, place the tape 0.5–1.0 cm above the navel level around a circle to measure. At the same time, the participants were required to stand with light garments and breathe naturally. The hip circumference was measured around the buttocks at the widest point [26]. We obtained blood samples from the antecubital vein of subjects in the morning, after an overnight fasting for at least eight hours. The tubes used to preserve blood samples contained EDTA [27]. A Bayer ContourTS blood glucose meter and test strips were used to measure fasting glucose levels.

### 2.5. Trace Elements in Hair

Hair roots were obtained from different parts of subjects. Samples were washed three times (15 min per wash) with detergent, and then washed three times with deionized water. After being degreased in acetone solution, the hair received natural drying. The hair was cut into segments of 1–2 mm, accurately weighed (0.100 ± 0.003 gram), and then put into a clean test tube, to which 1 mL of 70% HNO_3_ was added. The capped tube was placed into an electric digestion at 140 °C till the samples were completely digested, and then it was cooled at room temperature. Finally, the samples were diluted with high-purity deionized water to a constant volume of 20 mL with mixing. Inductively coupled plasma mass spectrometry was used to determine the content of trace elements in the hair.

### 2.6. Definitions

BMI was considered as a substitute for the degree of obesity [28]. According to the Chinese guidelines [29], normal weight, overweight, and obesity were defined by the value of BMI (normal weight: BMI < 24.0 kg/m^2^; overweight: 24.0 ≤ BMI ≤ 27.9 kg/m^2^; obesity: BMI > 28.0 kg/m^2^). This standard was used to subgroup subjects. In addition, abdominal obesity was determined by waist-hip ratios, defined as a waist-hip ratio ≥ 0.90 in men or ≥ 0.85 in women [30]. 

According to the diagnostic criteria of the WHO Expert Committee on Diabetes Mellitus, a normal fasting serum glucose level ranges from 3.9 mmol/L to 6.0 mmol/L. IFG means that a fasting serum glucose level ranges from 6.1 mmol/L to 6.9 mmol/L Self-reported DM and/or a fasting serum glucose level ≥7.0 mmol/L was regarded as DM. A family history of diabetes was defined as that there was at least one diabetic parent in the family [26]. 

Current smokers were defined as those who smoked at least one cigarette a day on average continuously for nearly 30 days. Alcohol drinking was defined as drinking alcohol once a week, past drinking and never drinking were all considered to be no drinking. “Often” referred to more than three times a week, “sometimes” referred to no more than three times a week, “rarely or never” referred to no more than once a week.

### 2.7. Data Analysis

Epidata 3.1 was used to establish the database, and the SPSS 17.0 statistical software was used for data analysis. The continuous variables were described as $\bar{X}$± SD (mean ± standard deviation) if they were in normal distributions. Alternatively, *M and Q* (median and interquartile）were used to describe the central tendency and the dispersion trend of measurements that did not follow the normal distribution. The least significant difference (LSD) procedure and the Dunnett’s T_3_ method in one way analysis of variance (ANOV) were used to compare the means of three groups of measurements with homogeneity or heterogeneity of variances (the adjusted α = 0.017), while the Kruskal Wallis Test (the adjusted α = 0.017) was used to compare hair metallic element contents and blood glucose levels (not in normal distribution) among three groups. Correlation between two variables was analyzed using the Spearman rank correlation. To analyze the influence of potential risk factors on IFG and diabetes with normoglycemic individuals as the reference group, we used polytomous logistic regression analysis, with adjustment for age, sex, family history of diabetes and BMI. A two-tailed *p* value < 0.05 was considered to be statistically significant.

## 3. Results

The valuation of different factors is shown in Table 1.

**Table 1:** The valuation of factors.

Factors	Variables	Valuation
Area	Area	Jingyu County = 1, Dongliao County = 2, Changling County = 3
Age	Age	≤29 = 1, 30~ = 2, 40~ = 3, 50~ = 4, 60~ = 5, ≥70 = 6
Sex	Sex	Male = 1, Female = 2
Smoking	Smoking	Current smokers = 1, Past smokers = 2, Never = 3
Alcohol drinking	Alcohol drinking	Yes = 1, No = 2
Diet Fruit	Diet Fruit	Eating meat more = 1, Eating vegetables more = 2, Appropriate = 3 Often = 1, Sometimes = 2, Rarely or never = 3
Exercise	Exercise	Often = 1, Sometimes = 2, Never or rare = 3
DM history	DM history	No = 0, Yes = 1
BMI	BMI	<18.5 = 1, 18.5~ = 2, 24.0~ = 3, 28.0~ = 4
Abdominal obesity	Abdominal obesity	No = 0, Yes = 1

525 subjects (including 332 males and 193 females), aged 18–77 years old were included in the present study. As shown in Table 2, age, weight, waist and hip sizes, and BMI were all significantly different between IFG or DM and normoglycemic individuals (*p* < 0.017). There was no significant difference in height among the three different groups (*p* > 0.017). 

**Table 2:** Characteristics of subjects with normal/impaired fasting glucose levels or diabetes.

Variables	Normoglycemic Levels (n = 391)	Impaired Fasting Glucose Levels (n = 89)	Diabetes (n = 45)
Age (year)	46.93 ± 12.70	52.22 ± 12.13 *	55.42 ± 11.01 *
Height (cm)	163.99 ± 8.63	165.06 ± 7.71	165.20 ± 8.81
Weight (kg)	63.65 ± 10.58	69.95 ± 10.46 *	70.52 ± 15.45 *
Waist (cm)	79.51 ± 9.97	86.01 ± 7.81 *	90.30 ± 13.67 *
Hip (cm)	93.32 ± 6.87	95.82 ± 6.02 *	98.07 ± 10.36 *
BMI (kg/m^2^)	23.66 ± 3.47	25.63 ± 3.05 *	25.78 ± 4.86

* *p* < 0.017, Comparisons between IFG/DM and normoglycemic individuals; There was no significance difference of all variables between DM and IFG subjects; *p* < 0.017: Statistically significant. Values were mean ± SD.

As displayed in Table 3, blood glucose levels and Cu/Zn ratios were significantly higher in IFG subjects or DM patients than that in normoglycemic individuals (glucose: *p* < 0.001 (IFG) and *p* < 0.001 (DM); Cu/Zn: *p* = 0.005 (IFG) and *p* = 0.001 (DM)). The content of Ca in IFG subjects or the content of Zn in IFG/DM patients was significantly lower than that in normoglycemic individuals (Ca: *p* = 0.011 (IFG); Zn: *p* = 0.015 (IFG); *p* < 0.001 (DM)). In contrast, DM patients had a significantly higher content of K and Na than normoglycemic individuals (*p* < 0.001 (K) and *p* = 0.002 (Na)). Significant differences in blood glucose levels and the content of K between DM patients and IFG subjects (*p* < 0.001 (glucose) and *p* = 0.008 (K)) were also observed. However, we did not find significant differences in the content of Cu, Se, Fe, and Mg among the three groups.

**Table 3:** Comparison of hair element contents and blood glucose levels among normoglycemic, IFG, and DM subjects.

Variables	Normoglycemic Individuals (n = 391)	Subjects with Impaired Fasting Glucose (n = 89)	Diabetes Patients (n = 45)
Blood glucose (mmol/L)	5.20 ± 0.90	6.40 ± 0.55^a^	8.40 ± 3.85 ^a,b^
Cu (μg/g)	9.72 ± 3.78	10.19 ± 4.77	9.80 ± 4.43
Se (μg/g)	0.01 ± 0.18	0.06 ± 0.20	0
Fe (μg/g)	68.97 ± 73.88	59.67 ± 50.65	73.05 ± 61.27
Zn (μg/g)	145.75 ± 63.73	136.68 ± 68.27 ^a^	122.29 ± 48.02 ^a^
K (μg/g)	285.40 ± 567.40	353.00 ± 641.00	693.20 ± 993.20 ^a,b^
Ca (μg/g)	817.20 ± 689.40	661.80 ± 579.60 ^a^	694.80 ± 565.60
Na (μg/g)	524.80 ± 936.00	562.20 ± 1247.10	834.00 ± 1276.40 ^a^
Mg (μg/g)	107.40 ± 100.99	101.00 ± 83.00	96.00 ± 68.50
Cu/Zn	0.07 ± 0.04	0.08 ± 0.04 ^a^	0.09 ± 0.06 ^a^

^a^ *p* < 0.017, Comparisons between IFG/DM and normoglycemic individuals; ^b^ *p* < 0.017, Comparisons between DM and IFG subjects; *p* < 0.017: Statistically significant. Kruskal Wallis Test, values are median ± Q.

The correlation of blood glucose levels and hair element contents is shown in Table 4. Contents of hair Cu, Se and Na were positively correlated with blood glucose levels (Cu: *r*_s_ = 0.135, *p* = 0.002; Se: *r*_s_ = 0.110, *p* = 0. 012; Na: *r*_s_ = 0.091, *p* = 0.038). 

**Table 4:** Correlation between blood glucose levels and hair element contents.

		Cu	Se	Fe	Zn	K	Ca	Na	Mg
Blood glucose	*r*_s_ *p*	0.135 * 0.002 *	0.110 * 0.012 *	−0.078 0 .076	−0.084 0.054	0.074 0 .090	−0.071 0.103	0.091 * 0.038 *	0.001 0.986

* The correlation was considered to be significant if *p* is less than 0.05 (2-tailed).

A polytomous logistic regression analysis was conducted to analyze the effect of various factors on susceptibility to IFG or DM compared with normoglycemic individuals as the reference group and age, sex, family history of diabetes, and BMI as covariates. As shown in Table 5, when other factors were constant, subjects who had a higher BMI were more likely to develop IFG or DM (IFG: OR = 1.15, OR 95% CI = 1.02–1.29; DM: OR = 1.15, OR 95% CI = 1.01–1.33). Moreover, rarely or never eating fruits was a risk factor for DM but not for IFG (DM: OR = 5.46, OR 95% CI = 1.87–15.98; IFG: OR = 1.70, OR 95% CI = 0.72–4.02). Subjects with abdominal obesity or DM history were more susceptible to DM (abdominal obesity: OR = 2.99, OR 95% CI = 1.07–8.37; DM history: OR = 2.69, OR 95% CI = 1.01–7.20). The risk effect of these factors was even stronger before adjusting for age, sex, family history of diabetes, and BMI in subjects (rarely or never eating fruits: OR = 6.72, 95% CI = 2.81–16.07; abdominal obesity: OR = 7.00, 95% CI = 3.24–15.13). We found that subjects living in Changling County had a significantly lower chance to suffer from IFG (OR and 95%CI for OR: 0.25, 0.08–0.74). The protective association for IFG was even stronger without controlling for age, sex, family history of diabetes, and BMI in subjects (OR: 0.18, 95% CI: 0.08–0.40). Living in Dongliao County was also found to be a protective factor for IFG before adjustment for these confounders (OR: 0.53, 95% CI: 0.28–0.98). Men and 60–69 years old subjects were found to be risk factors for IFG, and these subjects had a significantly higher incidence of high blood glucose levels. The OR and 95% CI for OR were 3.51 (1.34–9.18) for men and 6.64 (1.36–32.47) for age 60–69. We did not find a significant association between some lifestyles (eating more meat, exercise or physical activity, smoking, and alcohol drinking) and the content of various metallic elements (Fe, Zn, K, Ca, Na, and Mg) in subjects with IFG or DM.

**Table 5:** Polytomous logistic regression analysis of factors influencing impaired glucose metabolism.

Variables	IFG (n = 89)							DM (n = 45)						
	PR (%)	*p*		OR		95% CI		PR (%)		*p*		OR		95% CI
Zn		0.045		0.99		0.98,1.00				0.046		0.99		0.98,1.00
K		0.037		1.00		1.00,1.00				0.891		1.00		1.00,1.00
**BMI**		**0.025**		**1.15**		**1.02,1.29**				**0.042**		**1.15**		**1.01,1.33**
Fruit														
Often	52.8			1.00				26.7				1.00		
Sometimes	25.8	0.643		1.23		0.51,2.98		26.7		0.545		1.43		0.45,4.54
**Rarely or never**	21.3	0.224		1.70		0.72,4.02		46.7		**0.002**		**5.46**		**1.87,15.98**
Diet														
Eating more meat	15.7			1.00				6.7				1.00		
Eating more vegetables	23.6	0.084		0.36		0.11,1.15		35.6		0.116		4.13		0.71,24.15
Appropriate	60.7	0.219		0.57		0.23,1.40		57.8		0.195		2.93		0.58,14.83
Area														
Jingyu County	59.6			1.00				37.8				1.00		
Dongliao County	27.0	0.650		1.21		0.53,2.79		24.4		0.616		0.74		0.23,2.37
**Changling County**	13.5	**0.013**		**0.25**		**0.08,0.74**		37.8		0.697		0.80		0.27,2.42
Exercise														
Often	19.1			1.00				33.3				1.00		
Sometimes	22.5	0.193		2.09		0.69,6.37		8.9		0.547		0.65		0.16,2.63
Rarely or never	58.4	0.718		0.85		0.34,2.08		57.8		0.186		0.51		0.19,1.38
Smoking														
Current smokers	38.2			1.00				33.3				1.00		
Past smokers	13.5	0.805		1.18		0.32,4.27		17.8		0.794		1.20		0.30,4.83
never	48.3	0.759		0.87		0.37,2.06		48.9		0.673		0.79		0.27,2.33
Alcohol drinking														
Yes	43.8		1.00				26.7				1.00			
No	56.2	0.812	1.10		0.51,2.36		73.3		0.201		1.94		0.70,5.32	
Abdominal obesity														
No	41.6		1.00				26.7				1.00			
**Yes**	58.4	0.081	2.04		0.92,4.54		73.3		**0.037**		**2.99**		**1.07,8.37**	
Age														
≤29	4.5		1.00				2.2				1.00			
30~	10.1	0.807	1.21		0.27,5.43		6.7		0.497		2.67		0.16,45.33	
40~	23.6	0.465	1.71		0.40,7.22		22.2		0.182		6.08		0.43,86.12	
50~	37.1	0.076	3.69		0.87,15.62		26.7		0.227		5.24		0.36,77.31	
**60~**	18.0	**0.019**	**6.64**		**1.36,32.47**		35.6		0.159		7.39		0.46,119.12	
≥70	6.7	0.094	7.41		0.71,77.23		6.7		0.390		4.95		0.13,189.83	
Sex														
Female	29.2		1.00				37.8				1.00			
**male**	70.8	**0.010**	**3.51**		**1.34,9.18**		62.2		0.088		2.75		0.86,8.81	
DM history														
No	77.8		1.00				64.7				1.00			
**Yes**	22.2	0.188	1.77		0.76,4.14		35.3		**0.048**		**2.69**		**1.01,7.20**	

***OR*:** odds ratio, ***CI***: confidence interval, *p* values less than 0.05 are highlighted in bold.

## 4. Discussion

In this study, we investigated the influence of potential risk factors on IFG and Diabetes. These factors included demographic characteristics and health-related behaviors, body measurements, and laboratory measurements. Age, weight, waist and hip sizes, and BMI as well as the content of hair metallic elements such as Cu/Zn were significantly different between IFG/Diabetes patients and normal subjects. There were significant differences in blood glucose levels among three groups. The content of Ca in IFG subjects and Zn in DM patients was significantly lower than that in normoglycemic individuals, while the content of K and Na in DM patients was significantly higher when compared to that of normoglycemic individuals. Significant differences in the content of K between DM patients and IFG subjects were also observed.

Epidemiological studies have indicated that age, overweight, obesity, drinking, and smoking contribute to both IFG and DM [31,32,33,34]. This is consistent with our findings. Nevertheless, we failed to find a significant correlation between drinking or smoking and DM or IFG. It may be due to race or environmental differences. Also, a few studies found that overweight and obesity were independent risk factors for both IFG and DM [35,36,37]. Two nationwide cohort studies conducted in obese children and adolescents demonstrated that IFG risk was positively correlated with age and obesity [38]. Another study investigated the determinants of IFG in a population of Taiwan and showed that the likelihood of IFG was increased by 1.03 for each additional year of life, and subjects who were overweight or obese exhibited a higher probability of IFG [39]. Our study also found that men and 60–69 years old subjects had a significantly higher incidence of IFG [38,40,41,42]. Additionally, subjects with a higher BMI were more likely to develop IFG and Diabetes compared to subjects with the normal weight. Our results suggested that there might be a link between BMI and age, and a greater BMI and an older age might have a combined effect on IFG/DM.

Different from the findings of published studies [43,44], we did not observe a significant correlation between eating habits or exercise and the likelihood of IFG or DM. A study with middle-aged and senior subjects from Baoding, China and other studies on the relation between lifestyle and IFG/DM found that risk factors, such as sex, age, fruit intake, BMI, waist hip rate (WHR), waist circumference (WC) and DM family history, were significantly associated with abnormal glucose regulation, and eating more fruits and the female sex were protective factors [17,18,19,39]. We have come to similar conclusions. Studies have suggested that subjects with DM history and abdominal obesity were more likely to suffer from DM [39,44]. This view is consistent with our conclusion. There is evidence suggesting that increased fruit intake is also associated with less cardiovascular diseases and lower BMI in Chinese with Type 2 diabetes mellitus [45]. A meta-analysis of prospective cohort studies showed that higher fruit intake contributed to a significantly reduced risk of Diabetes [46]. In this study, we came to a similar conclusion that rarely or never eating fruits was a risk factor for Diabetes. By polytomous logistic regression analysis, we did not find significant associations of some lifestyle (such as eating more meat, physical exercise situation, smoking, or alcohol drinking) and the content of various metallic elements (Fe, Zn, K, Ca, Na, and Mg) with IFG or DM. Further studies on the potential impact of imbalanced elements levels on impaired glucose metabolism are necessary. We observed that subjects lived in Changling County did not have abnormal glucose levels compared to subjects who lived in other two places. This may be due to different lifestyles and mineral elements in diet in different regions [39]. 

The balance of elements in human body is affected by either absolute or relative deficiency of insulin secretion, and mineral elements in diet play an important role in the synthesis, secretion, and storage of insulin and energy metabolism [47,48,49]. To understand the relevance of various hair metallic elements and IFG/DM, a study of the relationship between dietary composition and the contents of hair metallic elements in a population from Taiwan showed that higher intakes of Ca, Mg, and total or non-heme iron were protective to IFG/diabetes [44]. Other studies showed that the mean values of Zn in serum were significantly lower in patients with diabetes than in control subjects [47,48,49]. Many studies have found that the content of Cu in diabetic patients was significantly higher than in healthy subjects [45,50]. In this study, we found that the contents of Cu, Se, and Na were positively correlated with blood glucose levels. The comparison among three groups showed that Ca and Zn may be protective factors for IFG or DM, and K, Na, and Cu/Zn were potential risk factors. However, we failed to come to the same conclusion in polytomous logistic regression analysis of factor influencing impaired glucose metabolism. There were also studies demonstrating that excessive iron content increased the severity of diabetes [50]. Furthermore, iron overload was closely associated with insulin resistance [46,51]. This may be due to the direct deposition of iron in damaged pancreatic cells, affecting the secretion of insulin [52]. Iron overload or deficiency could also result in obesity-related inflammation, hypoxia, and insulin resistance [53]. This difference may be due to the reason that the concentration of trace element is influenced by many factors, such as diet, time of blood sampling, and movement state. We found that the Cu/Zn ratio was significantly higher in the patients with IFG or T2D than in normal control subjects. Similar results were reported by others [20]. In addition, these studies showed that the serum Cu level was positively associated with HbA1c in T2D subjects. 

The mechanism of elevated serum Cu levels in diabetic patients is not clear. Some studies provided evidence that a specific proportional relationship exists between serum Cu and Zn, and that when subjects are suffered from IFG or diabetes, the proportional relationship of Cu and Zn was destroyed [50,54]. This may be due to the disorder of Cu use and metabolism in the body, or the mutual antagonism between Zn and Cu [55]. Increased Cu content causes the body to lose Zn, and reducing the content of Zn can lead to diabetes [50]. 

In conclusion, an abundance of data showed that age, weight, BMI and some health-related behaviors are associated with IFG and DM [17]. Lifestyle intervention or modification in subjects at high risk of DM has been proven effective in reducing and/or delaying the incidence of DM [18,19].

In the future preventive programs including lifestyle improvement, weight control and increased physical activity should be established for the large proportion of subjects at risk of DM. We also believe that an individual approach, as exemplified by a small weight loss, eating more fruit, or increasing physical activity in a modest way, will benefit subjects at risk of DM [56,57]. 

## 5. Limitations and Strengths

The polytomous logistic regression analysis in our study showed that the content of Zn had a protective association with IFG and DM (IFG: *p* = 0.045, OR = 0.99, 95% CI = 0.98–1.00; DM: *p* = 0.046, OR = 0.99, 95% CI = 0.98–1.00). The ORs did not reach a statistical significance. This is most likely due to the small number of cases. Therefore, further studies with larger study populations are needed to confirm our findings.

Some studies showed that in the conventional therapy of diabetic patients, the effect of trace element should be taken into account [58]. Supplementing trace elements from food appears to be the most effective way. The limitation of our study is that it did not elaborate a specific dietary composition. We hope to make further efforts to study nutrition and chronic diseases among residents in Jilin Province in the near future. To date, very few published studies have examined the relationship between trace elements and diabetes, although it has long been proposed that trace elements may be associated with diabetes. Therefore, the findings from the present study could help advance our understanding about the effects of trace elements on diabetes.

## 6. Conclusions

We selected subjects from representative regions (low prevalence of diabetes and/or considering the sampling results) of Jilin Province of Northeast China. And we found that living in Changling County, men and 60–69 years old subjects were risk factors for IFG. Less or no fruit eating, DM family history, abdominal obesity conferred vulnerability to DM. Subjects with high BMI were more likely to develop IFG and DM. In the future preventive programs including lifestyle improvement, weight control and increased physical activity should be established for the large proportion of subjects at risk of DM.
